# The medium-term impact of a micronutrient powder intervention on anemia among young children in Rural China

**DOI:** 10.1186/s12889-024-17895-2

**Published:** 2024-02-10

**Authors:** Siqi Zhang, Lei Wang, Renfu Luo, Scott Rozelle, Sean Sylvia

**Affiliations:** 1 International Business School, Shaanxi Normal University, Xi’an, China; 2 Center for Agricultural Policy, School of Advanced Agricultural Sciences, Peking University, Beijing, China; 3 Stanford Center On China’s Economy and Institutions, Stanford University, Stanford, CA USA; 4 Department of Health Policy and Management, University of North Carolina at Chapel Hill, Chapel Hill, NC USA; 5 Carolina Population Center, University of North Carolina at Chapel Hill, Chapel Hill, NC USA

**Keywords:** Anemia, Micronutrient powders, Early childhood, Rural China

## Abstract

**Background:**

Poor development of young children is a common issue in developing countries and it is well established that iron deficiency anemia is one of the risk factors. Research has shown that iron deficiency is a common micronutrient deficiency among children in rural China and can result in anemia. A previous paper using data from the same trial as those used in the current study, but conducted when sample children were younger, found that after 6 months of providing caregivers of children 6–11 months of age free access to iron-rich micronutrient powder (MNP) increased the hemoglobin concentrations (Hb) of their children. However, no effects were found 12 and 18 months after the intervention. The current study followed up the children four years after the start of the original intervention (when the children were 4–5 years old) and aims to assess the medium-term impacts of the MNP program on the nutritional status of the sample pre-school-aged children, including their levels of Hb, the prevalence of anemia, and the dietary diversity of the diets of the children.

**Methods:**

At baseline, this study sampled 1,802 children aged 6–11 months in rural Western China. The intervention lasted 18 months. In this medium-term follow-up study that successfully followed 81% (*n* = 1,464) of children (aged 49–65 months) from the original study population 4 years after the start of the intervention, we used both intention-to-treat (ITT) effect and average treatment on the treated effect (ATT) analyses to assess the medium-term impacts of the MNP distribution program on the nutritional status of sample children.

**Results:**

The ITT analysis shows that the MNP intervention decreased the prevalence of anemia of young children in the medium run by 8% (4 percentage points, *p* < 0.1). The ATT analysis shows that consuming 100 (out of 540) MNP sachets during the initial intervention led to a decrease in anemia of 4% (2 percentage points, *p* < 0.1). Among children with moderate anemia at baseline (Hb < 100 g/L), the intervention reduced the probability of anemia by 45% (9 percentage points, *p* < 0.1), and, for those families that complied by consuming 100 (out of 540) sachets, a 25% (5 percentage points, *p* < 0.05) reduction in the anemia rate was found. The MNP intervention also led to a persistent increase in dietary diversity among children that were moderately anemic at baseline. The results from the quantile treatment effect analysis demonstrated that children with lower Hb levels at baseline benefited relatively more from the MNP intervention.

**Conclusions:**

The findings of the current study reveal that the MNP intervention has medium-term effects on the nutritional status of children in rural China. The impacts of the MNP program were relatively higher for children that initially had more severe anemia levels. Hence, the implications of this study are that programs that aim to increase caregiver knowledge of nutrition and improve their feeding practices should be encouraged across rural China. Families, policymakers, and China’s society overall need to continue to pay more attention to problems of childhood anemia in rural areas. This is particularly crucial for families with moderately anemic children at an early age as it can significantly contribute to improving the anemia status of children across rural areas of China.

**Trial registration:**

ISRCTN44149146 (15/04/2013).

**Supplementary Information:**

The online version contains supplementary material available at 10.1186/s12889-024-17895-2.

## Background

In the developing world, more than 200 million children under 5 years of age are not fulfilling their developmental potential [1]. This study with data across 141 countries of the globe showed that, in 2010, the percentage of children at risk of poor development due to stunting or living in extreme poverty was 43% [1]. According to prior studies, one of the risk factors for developmental delays for young children in developing countries is iron deficiency anemia [2–4]. As of 2019, the World Health Organization (WHO) estimated that, globally, 39.8% of children under 5 years of age were anemic, a number that was only 2.6 percentage points lower in the previous decade [5]. A study with representative data across 133 countries also showed that 40% of children aged 6–59 months were anemic in 2019, only a small decrease from 48% in 2000 [6]. In China, iron deficiency anemia stands as the most prevalent risk factor for developmental delays among children, affecting approximately 30–40% of children under 2 years of age in rural areas nationwide [7–9]. According to the findings of the 2010–2013 China National Nutrition and Health Survey (CNNHS), the ratios of meeting the daily dietary iron intake for children aged 6–11 months and 12–23 months were 46.8% and 66.8%, respectively [10]. In poor, rural, Western regions of China, the rates of anemia among infants and toddlers under 12 months of age are even higher, up to 50% [7, 11].

Iron deficiency anemia in early childhood has both short-term and long-term consequences. Immediate consequences of anemia include cognitive, physical, and social-emotional delays [1, 7, 12–17]. A meta-analysis of five geographically diverse studies in Europe, Latin America, the Middle East, and North America estimated that, for each 10 g/L decrease in Hb in infancy, Intelligence Quotient (IQ) in early childhood (2–3 years later) decreased by 1.73 points [18]. Longer-term consequences include lower educational attainment, lower-quality employment, and reduced earnings in adulthood [19–21].

Multiple randomized trials have shown that home fortification using MNP can effectively raise iron intake and reduce anemia among children in the short term, especially for children with moderate to severe anemia [22–29]. A review of the literature that included 24 trials involving 33,147 children in low- and middle-income countries revealed that providing MNPs to children aged 6 to 23 months reduced the risk of anemia by 18% and lowered levels of iron deficiency by 53%, compared to children that did not receive any treatment [30].

Research in China has also found positive short-term impacts of MNP interventions on the prevalence of anemia among rural children, especially in children with moderate or severe anemia [11]. Wang et al. (2011) reported that an MNP intervention among children aged 6 to 29 months in rural Sichuan Province increased hemoglobin concentrations by 15.1 g/L and reduced the prevalence of anemia from 77 to 31%. In a follow-up survey conducted 15 months after the launch of the intervention, the prevalence of moderate anemia fell from 18 to 1% [11].

As cited above, the literature’s consensus—both internationally and in rural China—is that when households in the MNP program comply by providing their young children with MNP supplements, anemia rates will fall. Beginning with a pilot in 2012, China’s government launched a national program aiming to distribute one MNP sachet per day to children aged 6–24 months through local clinics. This program later expanded to 300 nationally-designated poverty counties in 2013. Although the implementation varied in intensity across regions, the program made free MNP sachets available to approximately 400,000 children across rural China at a cost of USD 43 million (RMB 300 million) in 2013. By 2019, this program covered 9.47 million rural children from 832 counties in 22 provinces across China. However, it is important to note that the national MNP program in China ends when children are 24 months [31]. Although most studies of the prevalence of anemia in rural China were carried out before the implementation of the national MNP program, many studies found relatively high rates (ranging from 19%-32.7%) of anemia among older children aged 3 to 6 years, including preschool-aged children [32–34]. A natural question, therefore arises, given the documented negative impacts of anemia on health and learning outcomes of children that are preschool age [35]: Are there any medium- or long-term impacts (e.g., in the context of this paper, on children that are preschool age) of MNP programs that enrolled children only until they were 24 months?

In recent years, the literature has begun to focus on issues regarding the dynamic complementarities of programs in early childhood development. Dynamic complementarities refer to the better health (or higher skills) that are produced in one period that are found to lead to more favorable outcomes (e.g., better health/higher skills) in subsequent periods (i.e., when children are older). If dynamic complementarities are strong, the short-term impact of a program may persist or become stronger in the long-term. Although few studies have shown that supplementation intervention programs can improve health outcomes in children later in life [36, 37], the nature of the dynamic complementarities and the medium- and long-term impacts on children’s anemia status of interventions, such as China’s MNP program, are not clear. For example, in a paper by Alderman et al. (2014), the authors directly call for more studies on the dynamic complementarities of nutrition programs for young children, claiming that currently, there are studies that suggest they could be positive, neutral, or even negative [38].

The aim of this paper is to examine if there are medium-term impacts of an MNP intervention on children’s nutritional status in rural China. To achieve this goal, this study reports the medium-term results of an intervention that involved the distribution of MNPs with iron to the caregivers of young children. This evaluation was initially designed to inform policymakers about the potential impacts of the forthcoming national program. The data in this paper were collected as part of the evaluation of a cluster-randomized controlled trial (RCT) that was examining the impact of an MNP intervention on young children in rural China; the results of the evaluation was originally reported in Luo et al. (2017) [7]. Beginning with a pilot in 2012, and later expanded to 300 nationally designated poverty counties in 2013, the national program aims to distribute one MNP sachet per day to children aged 6–24 months through local clinics [39]. Although implementation varied across regions, this program made free MNP sachets available to approximately 400,000 children across rural China at a cost of USD 43 million (RMB 300 million) in 2013 [39]. By 2019, this program covered 9.47 million rural children from 832 counties in 22 provinces across China.

In a trial conducted prior to the rollout of the policy that tested the effects of MNP distribution to caregivers of young children, it was found that delivering MNP sachets to households led to an improvement in hemoglobin levels after six months of the program (when children were 12–17 months old) but that effects were not detectable by the time children were 24–29 months old [7]. Even though the effects of the intervention faded out in the short-term, it is possible that such effects would come out in the longer-term, either through the mechanism of the enhancement of parental health knowledge or the improvement of parental feeding practices [37, 40–42]. Unfortunately, to our knowledge, no study in rural China has ever investigated the effects of the MNP intervention in the longer term. Here, this study analyzes the medium-run effects of the program on anemia rates and feeding practices among these children four years after the start of the initial intervention (around two years after the end of the MNP distribution).

This study contributes to the existing literature by providing new evidence on the medium-term impacts of an MNP intervention program in rural China. To our knowledge this is the first study to examine the medium-term effects of an MNP intervention on child nutritional status in rural China, a place where nearly 10% of the world’s children live and grow up. Another strength of this study lies in its ability to draw causal conclusions as the underlying research utilizes results from a randomized controlled trial to measure the effect of the intervention on the anemia of young children in rural China. The study also is an effectiveness trial designed to mimic the medium-term impact of a government program.

## Methods

### Study design and participants

Data used in this study were drawn from a follow-up survey conducted four years after the start of a randomized-controlled trial that was originally designed to evaluate the impact of MNP intervention on young rural children in China. The trial was conducted in rural areas of the Qinba Mountain Area of Shaanxi Province. This area is a remote mountainous region prone to natural disasters and was known as a poverty-struck area throughout history. In 2013, the per capita gross domestic product (GDP) of the region was USD 1,219 (RMB 7,896), lower than the per capita net income of rural residents nationally (USD 1,373; RMB 8,898).

This study calculated the sample size for the study to detect a 0.2 standardized effect on the study’s main outcome. Based on previous studies [43–47], this study assumed an intracluster correlation of 0.1 and also assumed that baseline scores would account for 50% of the variation in scores at endline. We further assumed that there would be four sample children per village after attrition. Based on these parameters, this study calculated that the study required a sample with 112 villages/groups to detect a standardized effect of 0.2 at 80% power given a significance level of 0.05. We added five villages to each group to overpower the study and to account for potential attrition.

To select the baseline sample in 2013, the research team followed a multistage cluster sampling framework designed to yield a near representative sample of rural households in the study area. First, 11 nationally designated impoverished counties within the sample area were randomly selected for inclusion in the study. Next, all townships (the middle level of administration between county and village) within the 11 sample counties were included in the study, excluding the township in each county that housed the county seat, as well as any townships that did not have any villages with a population of at least 800. Two villages were then randomly selected within each township, and all children between 6 and 11 months of age were enrolled. We selected an additional 3 villages by randomly selecting 3 townships and 1 village in each selected township to meet power requirements. Children were enrolled in two cohorts: The first cohort enrolled children in April 2013, and the second cohort of children in the target age range was enrolled from the same sample villages in October 2013. Overall, the baseline sample included 1,802 children from 351 villages in 11 counties.

**Fig. 1:**
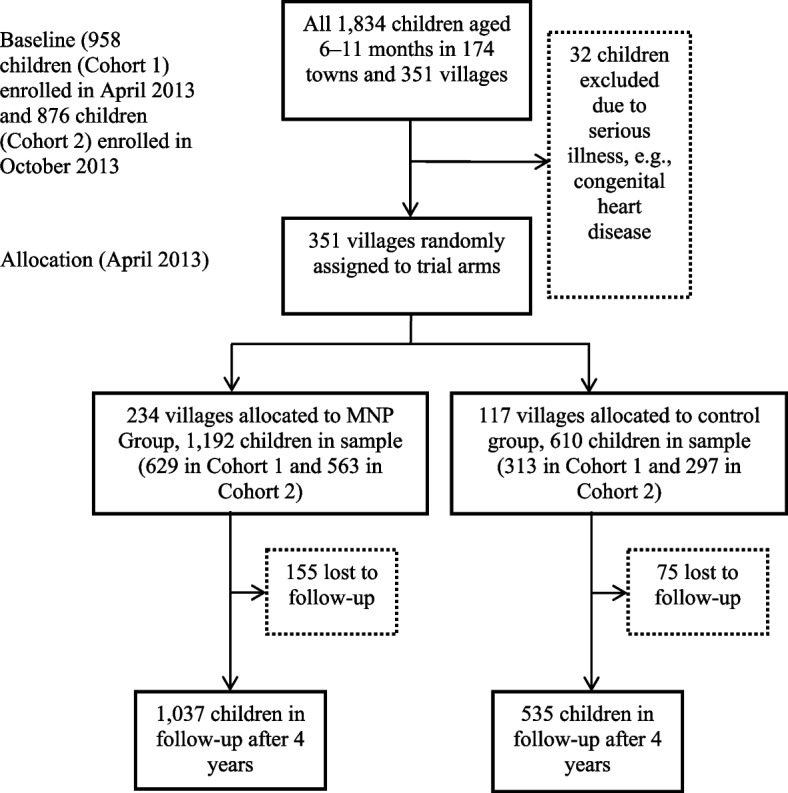
Experiment profile. Because 100 observations were missing data for the outcome variables, and because there were eight children who had severe anemia at baseline whom we excluded from the analysis, the number of observations in the final sample is 1,464

Of the 1,802 children and families in the baseline sample, this study was able to follow up with 1,572 children (when they were 4–5 years of age) and their families that were present in the survey wave of 2017 (Fig. 1). No villages (clusters) were lost. Because 100 observations were missed for the outcome variables (specifically, hemoglobin concentration, due to the fact that the sample caregivers refused to let the nursing/survey team conduct the testing) and because there were 8 children that had severe anemia at baseline that were excluded from the analysis, the number of observations in the final sample for this study is 1,464. This study team found no evidence that attrition was correlated with treatment assignment (see online Supplementary Table S1). The primary outcome variables and the secondary outcome variables were balanced in the treatment and control groups before the intervention started (see online Supplementary Table S2).

### Randomization and masking

Following the baseline survey (described below), this study randomly assigned 234 villages to the MNP intervention group and 117 villages to the control group, using computer code (in Stata version 12). Randomization was stratified within counties. The intervention period lasted for 18 months for both cohorts (from April 2013 to October 2014 for Cohort 1 and from October 2013 to April 2015 for Cohort 2). This study did not use a placebo in the control group for ethical and practical reasons. None of the caregivers, in either the treatment or control village, was aware that they were in a randomized control trial.

### Intervention

This study provided caregivers of all children in the treatment group with a free 6-month supply of MNP sachets every 6 months for a total of 18 months. During the initial distribution, this study also provided one-on-one health education training on nutrition and feeding practices to treatment caregivers. The training also included information on the causes and consequences of anemia. The team also provided oral and written instructions on how to administer the nutrition powder. Caregivers were instructed to provide one sachet per day mixed with the child’s food. In the treatment groups, enumerators also provided each household with a plastic storage envelope to store the NurtureMate packets, along with instructions to return the empty packets to the survey team every 6 months. Enumerators counted both unused and empty packets to evaluate compliance.

The MNP sachets used in this intervention were a Heinz-produced micronutrient powder called “NurtureMate.” This MNP is recommended for infants and toddlers aged 6–36 months. It is tasteless, and each daily-dose sachet contains a mix of iron (6 mg); zinc; vitamins A, C, D, B_1,_ B_2_, B_6_, B_12_; and folic acid. The full composition of the NurtureMate sachets was based on the general standard for complementary food supplements in China [48] and was approved for use in the national MNP program in 2012 [39]. It is recommended that caregivers give infants 5 packets per week or 1 packet per day.

### Measures

The baseline survey was conducted in April 2013 and October 2013 for Cohorts 1 and 2, respectively. Subsequent surveys were then conducted every six months for the duration of the 18-month intervention (2013 to 2015). A follow-up survey of both cohorts was conducted in June 2017, when children were 49–65 months (or 4–5 years) old. For each survey wave, this study recruited local college students to serve as enumerators for the surveys. Nurses from Xi’an Jiaotong Medical School conducted hemoglobin testing. All enumerators and nurses participated in a 15-day training program before each wave of data collection. Each survey wave collected three types of data, including data on child anemia status, child dietary diversity, and child and household characteristics. Our primary outcome variable is child anemia status, and the secondary outcome is child dietary diversity.

This study measured the hemoglobin concentration of each sampled child using a HemoCue HB 201 + finger-prick system (HemoCue Ltd., Ängelholm, Sweden). The nurses collected the second drop of capillary blood from the children for Hb measurement to enhance the data's accuracy. The primary outcome of the trial is anemia defined as cutoffs in Hb concentration, following WHO guidelines. Children aged 6–59 months with Hb < 110 g/L at sea level are defined as anemic. Mild, moderate, and severe anemia are defined as hemoglobin levels below 110, 100, and 70 g/L for children aged 6–59 months, and as hemoglobin levels below 115, 110, and 80 g/L for children aged 60–65 months [49]. Long-term residency at high altitudes (≥ 3,000 feet above sea level) causes a generalized upward shift in hemoglobin concentrations. Because many of the children in our sample reside mainly in high-altitude communities, this study adjusted the hemoglobin levels of concentration for anemia based on standards set by the Centers for Disease Control and Prevention (CDC) [50, 51]. Specifically, for children living at less than 3,000 feet above sea level, this study did not make any adjustments. For children living at 3,000–3,999 feet above sea level, this study reduced the levels of hemoglobin concentration by 2 g/L; for children living at 4,000–4,999 feet above sea level, reduced the levels of hemoglobin concentration by 3 g/L; for children living at 5,000–5,999 feet above sea level, reduced the levels of hemoglobin concentration by 5 g/L; for children living at 6,000–6,999 feet above sea level, reduced the levels of hemoglobin concentration by 7 g/L.

In each survey wave, this study collected information on the feeding practices used by the sample families, using guidelines for infant and child feeding, “Indicators for assessing infant and young child feeding practices,” set by the WHO [52, 53]. Each child’s primary caregiver was given a list of seven food types, including (a) grains, roots, and tubers; (b) legumes and nuts; (c) dairy products (milk, yogurt, and cheese); (d) flesh foods (meat, fish, poultry, and liver or organ meats); (e) eggs; (f) vitamin-A rich fruits and vegetables; and (g) other fruits and vegetables. They were then asked whether the child had eaten from each of the seven food groups during the previous day. For analysis of the data, this study used three alternative approaches to measure the feeding practices. Following previous research [54–56], the first approach was to use a dummy variable, minimum dietary diversity, that took a value of 1 if the child consumed from a minimum of four food groups each day, and 0 otherwise. This approach to measuring dietary diversity has been used in a number of other studies [7, 54–56]. Next, this study constructed a dietary diversity index (continuous variable) using a polychoric principal components method, which has been widely used in previous studies for variable reduction in the face of redundant or correlated exposures. In this sense, the results that use this approach will have clearer interpretability [57, 58]. Finally, using the same methodology and following the literature, this study constructed a calorie index to measure the level of caloric intake, an important determinant of a child’s nutritional status [59–61]. In this study, the calorie index was constructed based on whether the child consumed the following items on the day prior to the survey: (a) rice soup, porridge, noodle soup, buns, rice, or other grains; (b) fats (including oil used to stir-fry food); (c) cookies, desserts, sweets, chocolate, or other sweet food; (d) food with seasoning (hot pepper, garlic, ginger, etc.); (e) solid (e.g., rice, steamed buns), semi-solid (e.g., porridge); or (f) soft food (e.g., flour paste, fruit puree, mashed vegetables).

In addition to data on child anemia status and dietary diversity, this study collected data on child characteristics. Child characteristics included the child’s age in months, gender, and whether the child had a low birth weight. The age of each child was taken from the child’s birth certificate.

### Statistical analysis

Data analysis was performed using Stata 16.0 (Stata Corporation, College Station, TX, USA). Descriptive statistics were calculated, presenting continuous variables as means (standard deviation) and discrete variables as frequencies (%). Kernel density plots were drawn to show the probability distributions of child Hb levels at the baseline and follow-up survey. To examine the ITT effects of the intervention, ordinary least squares regressions were employed. Baseline covariates, including the child’s age in months, gender, whether the child had a low birth weight, were included. Cohort and county fixed effects were also included in the regression analyses. In all analyses, Huber-White cluster-adjusted standard errors were used to account for clustering within villages (the level of randomization). To assess the ATT effects of the intervention, an instrumental variable (IV) approach was used in order to detect the dose–response relationship between MNP sachet use and anemia status. To explore which type of children (those with different levels of anemia in terms of severity) were able to benefit from the intervention, we adopted a quantile treatment effect analysis which allowed us to evaluate the partial effects that the treatment exerted on different quantiles of the sample children. The significant levels for all analyses were set at *P* < 0.1 (two-tailed).

## Results

Of the 1,802 children and families in the baseline sample, this study were able to follow up with 1,572 children (when they were 4–5 years of age) and their families who were present in the survey wave of 2017 (Fig. 1). No villages (clusters) were lost. Because 100 observations were missed for the outcome variables (specifically, hemoglobin concentration, due to the fact that the sample caregivers refused to let the nursing/survey team conduct the testing) and because there were 8 children who had severe anemia at baseline whom we excluded from the analysis, the number of observations in the final sample for this study is 1,464. We find no evidence that attrition is correlated with treatment assignment (see online Supplementary Table S1). Our primary outcome variable and the secondary outcome are balanced in the treatment and control group before the intervention start (see online Supplementary Table S2).

### Average impact of the MNP intervention on child anemia status

**Fig. 2:**
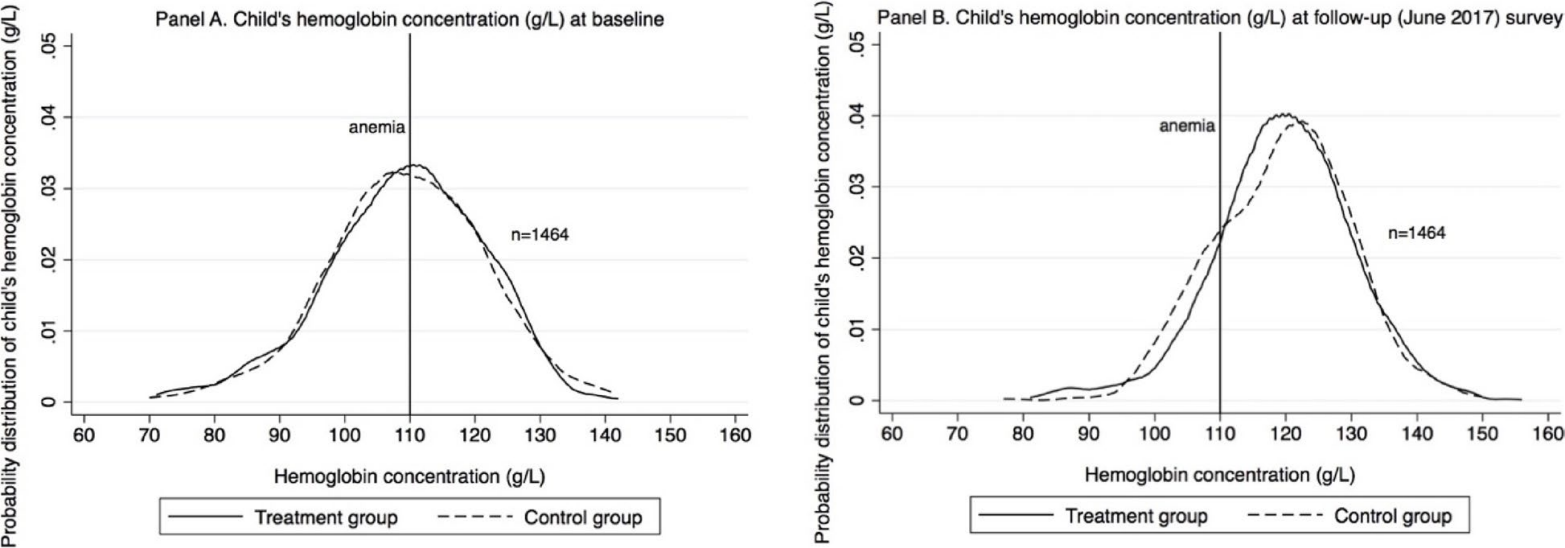
Probability distribution of child's hemoglobin concentration (g/L) at baseline and follow-up (June 2017) survey Anemia is defined as Hb < 110 g/L when child was 6–59 months old, and Hb < 115 g/L when child was 60–65 months old

Figure 2 shows the probability distribution of the hemoglobin concentrations of the sample children for the control and treatment groups at baseline in 2013 (Panel A) and follow-up survey in 2017 (Panel B). The distributions of the control and treatment groups overlap at baseline, with around 50% of children at < 110 g/L, the threshold for anemia. In the June 2017 follow-up four years later, both distributions have shifted to the right. The Kolmogorov–Smirnov test also showed that the differences in the levels of hemoglobin concentrations between treatment group and control group are not statistically significant (*p*-value = 0.489). However, there appears to be a larger number of children who fall below the anemia threshold in the control group.

Table 1 presents the results of the ITT analysis of the medium-term impacts of the MNP intervention on hemoglobin concentrations and anemia prevalence. The results show that the impact of the MNP intervention on hemoglobin concentrations for three sample groups were not statistically significant (full sample, anemic sample at baseline, and moderate anemic sample at baseline). In contrast, the results demonstrate that the MNP intervention significantly decreased anemia rates by 8% (4 percentage points, *p* < 0.1; Panel A, Row 1, Column 2) and 45% (9 percentage points, *p* < 0.1; Panel C, Row 7, Column 2) for the full sample and the moderate anemic sample at baseline, respectively. The deductions were calculated based on the prevalence at baseline (anemia prevalence was 49%, and moderate anemia prevalence was 20% at baseline) and the effect sizes of the intervention on anemia prevalence. The coefficients are significant at the 10% level.

**Table 1:** MNP program treatment effects on child anemic status 4 years after the start of the intervention (intention-to-treat analysis)

		Hemoglobin concentration (g/L) (95% CI)	Anemia prevalence (1 = yes) (95% CI)
Variable		(1)	(2)
Panel A: Full sample (*N* = 1,464)			
(1)	Treatment	0.34 (-0.97 to 1.64)	-0.04* (-0.09 to 0.01)
	(1 = yes)	(0.66)	(0.02)
(2)	Mean (SD) of outcome in the control group	119.44 (10.29)	0.21 (0.41)
(3)	Mean (SD) of outcome in the treatment group	119.66 (10.57)	0.17 (0.38)
Panel B: Anemic sample at baseline (*N* = 721)			
(4)	Treatment	0.26 (-1.40 to 1.92)	-0.03 (-0.09 to 0.03)
	(1 = yes)	(0.84)	(0.03)
(5)	Mean (SD) of outcome in the control group	118.80 (10.18)	0.22 (0.41)
(6)	Mean (SD) of outcome in the treatment group	118.91 (10.37)	0.19 (0.39)
Panel C: Moderate anemic sample at baseline (*N* = 289)			
(7)	Treatment	1.05 (-1.47 to 3.57)	-0.09* (-0.19 to 0.01)
	(1 = yes)	(1.28)	(0.05)
(8)	Mean (SD) of outcome in the control group	118.51 (10.86)	0.26 (0.44)
(9)	Mean (SD) of outcome in the treatment group	119.12 (11.05)	0.19 (0.39)

Anemic is defined as Hb < 110 g/L when child was 6–59 months old, and Hb < 115 g/L when child was 60–65 months old; moderate anemic is defined as 70 g/L < Hb < 100 g/L when child was 6–59 months old, and 80 g/L < Hb < 110 g/L when child was 60–65 months old. Each column presents the results of one regression of the outcome variable (corresponding to the column title) on the treatment dummy variable. Controls include the baseline value of the outcome variable, child’s age, gender, and whether the child had a low birth weight. We adjusted for cohort and county fixed effects, and standard errors are clustered at village level in full samples

^*^p < 0.10

### Dose–response effect of the MNP intervention on child anemia status

**Fig. 3:**
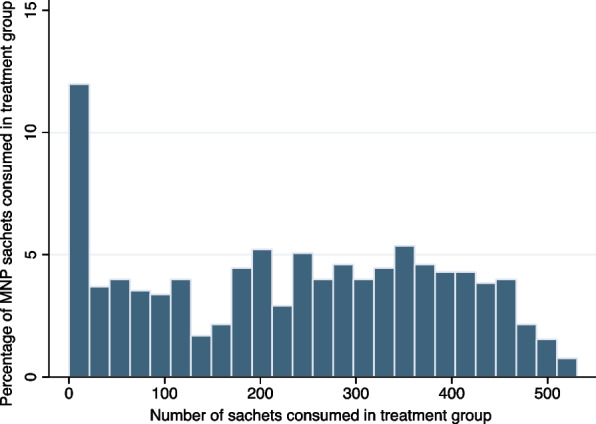
Percentage of MNP sachets consumed in treatment group

Figure 3 displays the percentage of MNP sachets consumed by sample children in the treatment group. As seen in the figure, a large share of the sample households/young children did not comply with the treatment. In every 6-month period of the 18-month MNP intervention, half of the children in the treatment group consumed approximately less than 50% of the total MNP sachets (see online Supplementary Tables S3, S4).

Table 2 shows the medium-term effects of the MNP intervention using the average treatment effect on the treated (ATT) model. Based on the data, it is estimated that, when a child consumed 100 MNP sachets during the 18 months of the program, this led to significant decreases in anemia by 4% (2 percentage points, *p* < 0.1; Panel A, Row 1, Column 2) and 25% (5 percentage points, *p* < 0.05; Panel C, Row 7, Column 2) for the full sample and the moderate anemic sample, respectively, at baseline. The results also show that the effect of the MNP intervention on the anemia rate was not statistically significantly different from zero for the anemic sample at baseline, and no significant treatment effects on child hemoglobin concentrations were found.

**Table 2:** Dose–response effects of per 100 MNP sachets consumption on child anemic status (average treatment effects on the treated analysis)

		Hemoglobin concentration (g/L) (95% CI)	Anemia prevalence (1 = yes) (95% CI)
Variable		(1)	(2)
Panel A: Full sample (*N* = 1,069)			
(1)	Effect of per 100 MNP sachet consumption	0.13 (-0.44 to 0.69)	-0.02* (-0.04 to 0.0015)
		(0.29)	(0.01)
(2)	Mean (SD) number of sachets consumed in the treatment group	238.13 (149.93)	
(3)	Median number of sachets consumed in the treatment group	247.5	
Panel B: Anemic sample at baseline (*N* = 524)			
(4)	Effect of per 100 MNP sachet consumption	0.15 (-0.60 to 0.91)	-0.02 (-0.05 to 0.007)
		(0.39)	(0.01)
(5)	Mean (SD) number of sachets consumed in the treatment group	233.03 (148.43)	
(6)	Median number of sachets consumed in the treatment group	242	
Panel C: Moderate anemic sample at baseline (*N* = 210)			
(7)	Effect of per 100 MNP sachet consumption	0.77 (-0.46 to 2.00)	-0.05** (-0.096 to -0.008)
		(0.63)	(0.02)
(8)	Mean (SD) number of sachets consumed in the treatment group	220.49 (144.33)	
(9)	Median number of sachets consumed in the treatment group	221.5	

Anemic is defined as Hb < 110 g/L when child was 6–59 months old, and Hb < 115 g/L when child was 60–65 months old; moderate anemic is defined as 70 g/L < Hb < 100 g/L when child was 6–59 months old, and 80 g/L < Hb < 110 g/L when child was 60–65 months old. Each column presents the results of one regression of the outcome variable (corresponding to the column title) on the treatment dummy variable. Controls include the baseline value of the outcome variable, child’s age, gender, and whether the child had a low birth weight. We adjusted for cohort and county fixed effects, and standard errors are clustered at village level in full samples. Data missing for 395 samples in regard to the number sachets consumed (244 samples in Cohort 1 and 151 samples in Cohort 2)

^*^p < 0.10

^**^p < 0.05

### Average impact of the MNP intervention on child dietary diversity

Table 3 presents the results of the ITT analysis of the impact of the MNP intervention (when they were 6–11 months old) on feeding of the children when they were 49–65 months old. The results show that the effect sizes on minimum dietary diversity, dietary diversity index, or calorie index were not significant for the full sample and the anemic sample at baseline (Panels A and B). For the moderate anemic sample at baseline, the ITT analysis demonstrates that the MNP intervention increased minimum dietary diversity and dietary diversity index by 11 percentage points (*p* < 0.1; Panel C, Row 7, Column 1) and 20 percentage points (*p* < 0.1; Panel C, Row 7, Column 2), respectively. The coefficients are significant at the 10% level.

**Table 3:** MNP program treatment effects on child dietary diversity 4 years after the start of the intervention (intention-to-treat analysis)

		Minimum dietary diversity (1 = 4 or more food groups) (95% CI)	Dietary diversity index (95% CI)	Calorie index (95% CI)
Variable		(1)	(2)	(3)
Panel A: Full sample (*N* = 1,464)				
(1)	Treatment	0.03 (-0.03 to 0.08)	-0.02 (-0.12 to 0.08)	-0.02 (-0.14 to 0.09)
	(1 = yes)	(0.03)	(0.05)	(0.06)
(2)	Mean (SD) of outcome in the control group	0.67 (0.47)	0.007 (1.00)	0.008 (1.00)
(3)	Mean (SD) of outcome in the treatment group	0.70 (0.46)	-0.001 (1.00)	-0.007 (1.00)
Panel B: Anemic sample at baseline (*N* = 721)				
(4)	Treatment	0.02 (-0.05 to 0.09)	-0.04 (-0.18 to 0.09)	0.06 (-0.11 to 0.22)
	(1 = yes)	(0.04)	(0.07)	(0.08)
(5)	Mean (SD) of outcome in the control group	0.69 (0.46)	0.05 (0.96)	-0.03 (1.05)
(6)	Mean (SD) of outcome in the treatment group	0.70 (0.46)	-0.01 (0.99)	0.01 (0.98)
Panel C: Moderate anemic sample at baseline (*N* = 289)				
(7)	Treatment	0.11* (-0.01 to 0.22)	0.20* (-0.03 to 0.43)	-0.13 (-0.37 to 0.11)
	(1 = yes)	(0.06)	(0.12)	(0.12)
(8)	Mean (SD) of outcome in the control group	0.63 (0.48)	-0.07 (1.02)	0.05 (0.99)
(9)	Mean (SD) of outcome in the treatment group	0.73 (0.44)	0.08 (0.96)	-0.01 (0.97)

Anemic is defined as Hb < 110 g/L when child was 6–59 months old, and Hb < 115 g/L when child was 60–65 months old; moderate anemic is defined as 70 g/L < Hb < 100 g/L when child was 6–59 months old, and 80 g/L < Hb < 110 g/L when child was 60–65 months old. Each column presents the results of one regression of the outcome variable (corresponding to the column title) on the treatment dummy variable. Controls include the baseline value of the outcome variable, child’s age, gender, and whether the child had a low birth weight. We adjusted for cohort and county fixed effects, and standard errors are clustered at village level in full samples

^*^p < 0.10

### Quantile Treatment Effects (QTE) analysis

Table 4 provides the quantile treatment effects of the MNP intervention on child hemoglobin concentration, the dietary diversity index, the calorie index, and the effects by different subgroups with different levels of anemic severity. The results in Panels A and B indicate that, for the full sample and anemic sample at baseline, the effect sizes on hemoglobin concentration, dietary diversity index, or calorie index were not significant at the 25th, 50th, or 75th percentiles. For the more vulnerable children, the results showed that the impact of intervention on hemoglobin concentration and dietary diversity index were significant at the 25th percentile (*p* < 0.05; Panel C, Row 9, Column 3) and 75th percentile (*p* < 0.05; Panel C, Row 10, Column 3), respectively. No heterogeneous effect, however, was found on calorie index at the 25th, 50th, or 75th percentiles (Panel C, Row 11, Columns 1–3).

**Table 4:** Quantile treatment effects of MNP program on child hemoglobin concentration, dietary diversity index, and calorie index

		25th (95% CI)	50th (95% CI)	75th (95% CI)
Variable		(1)	(2)	(3)
Panel A: Full sample (*N* = 1,464)				
		Hemoglobin concentration (g/L)		
(1)	Treatment	0.84 (-0.81 to 2.50)	-0.02 (-1.29 to 1.25)	-0.08 (-1.57 to 1.41)
	(1 = yes)	(0.84)	(0.65)	(0.76)
		Dietary diversity index		
(2)	Treatment	-0.04 (-0.21 to 0.14)	-0.03 (-0.20 to 0.14)	-0.03 (-0.16 to 0.09)
	(1 = yes)	(0.09)	(0.09)	(0.06)
		Calorie index		
(3)	Treatment	0.00 (-0.18 to 0.18)	0.00 (-0.25 to 0.25)	0.00 (-0.09 to 0.09)
	(1 = yes)	(0.09)	(0.13)	(0.00)
Panel B: Anemic sample at baseline (*N* = 721)				
		Hemoglobin concentration (g/L)		
(5)	Treatment	1.34 (-1.01 to 3.68)	0.62 (-1.07 to 2.32)	0.50 (-1.59 to 2.60)
	(1 = yes)	(1.19)	(0.86)	(1.07)
		Dietary diversity index		
(6)	Treatment	-0.03 (-0.25 to 0.18)	-0.09 (-0.31 to 0.14)	-0.00 (-0.20 to 0.19)
	(1 = yes)	(0.11)	(0.11)	(0.10)
		Calorie index		
(7)	Treatment	0.01 (-0.33 to 0.36)	-0.00 (-0.25 to 0.25)	0.00 (-0.29 to 0.29)
	(1 = yes)	(0.18)	(0.12)	(0.00)
Panel C: Moderate anemic sample at baseline (*N* = 289)				
		Hemoglobin concentration (g/L)		
(9)	Treatment	2.81** (0.03 to 5.58)	-0.10 (-3.28 to 3.07)	-0.73 (-4.24 to 2.79)
	(1 = yes)	(1.41)	(1.61)	(1.78)
		Dietary diversity index		
(10)	Treatment	0.20 (-0.16 to 0.55)	0.21 (-0.16 to 0.58)	0.29** (0.03 to 0.55)
	(1 = yes)	(0.18)	(0.19)	(0.13)
		Calorie index		
(11)	Treatment	-0.17 (-0.68 to 0.34)	-0.00 (-0.35 to 0.35)	-0.00 (-0.21 to 0.21)
	(1 = yes)	(0.26)	(0.18)	(0.01)

Anemic is defined as Hb < 110 g/L when child was 6–59 months old, and Hb < 115 g/L when child was 60–65 months old; moderate anemic is defined as 70 g/L < Hb < 100 g/L when child was 6–59 months old, and 80 g/L < Hb < 110 g/L when child was 60–65 months old. Each column presents the results of one regression of the outcome variable (corresponding to the column title) on the treatment dummy variable. Controls include the baseline value of the outcome variable, child’s age, gender, and whether the child had a low birth weight. We adjusted for cohort and county fixed effects, and standard errors are clustered at village level in full samples

^**^p < 0.05

## Discussion

This study uses a large-scale cluster randomized controlled trial of MNP in rural China and assesses the medium-term impact of the MNP intervention aimed at promoting young children’s health outcomes. The findings of this study show that, four years after the start of the intervention, when the sample children were 49–65 months old, the medium-term impact of the MNP intervention significantly decreased the prevalence of anemia and improved the dietary diversity of young children. Based on a large sample cluster randomized control trial, the findings are internally valid. In addition, the measures were valid, and the attrition was balanced in the treatment and control groups. The ATT estimated were much larger than the ITT estimation, which further emphasized the importance of compliance on the impact of MNP on child health status. The findings are consistent with the study by Lundeen et al. (2019). According to this study, four years after the initiation of a national Infant and Young Child Nutrition program, the prevalence of iron deficiency anemia was lower in the treatment group than that of the control group. The underlying reason for the reduction in iron deficiency anemia in a medium-term was that caregivers in the treatment group acquired more knowledge about feeding and nutrition and improved their feeding practices for the children, compared to caregivers in the control group [62].

The larger effects estimated by ATT than by ITT demonstrates that compliance remains a major challenge in the intervention. In fact, this is not new to the intervention in this study. Previous studies have shown that compliance is always a risk factor that can influence the effect size for in-home programs [63, 64]. In this sense, ensuring compliance is one of the keys to effective interventions in the future implementation of such program for rural China.

This study finds significant medium-term effects on the quality of the dietary diversity of the children in the treatment group. The results are consistent with previous research outside of China, which found that MNP interventions may lead to positive effects on the dietary diversity in the medium term (after 1.5 or 2 years of the start of the intervention) by increasing caregiver knowledge of nutrition and improving their feeding practices [40–42]. These results also illustrate the importance of MNP intervention for improving the anemia status of children not only directly but also by increasing the knowledge of nutrition of caregivers, which, in turn, appears to have improved caregiver feeding practices in the medium term.

The results of the heterogeneity analysis of the impact on child anemia status, minimum dietary diversity, the dietary diversity index, and the calorie index showed that the intervention did lead to an improvement of the Hb and dietary diversity indices of the children with lower hemoglobin levels (see online Supplementary Tables S5, S6, S7, S8, S9). The findings are consistent with the literature that suggests that children with lower hemoglobin levels benefit more from the MNP intervention [4, 17]. An anemia strategy review by the United Nations High Commission for Refugees found that the MNP intervention seems to have preferentially reduced the prevalence of severe and moderate anemia in children. In other words, if the baseline anemic situation of the children is poorer, more significant gains can be made [4]. These results are similar to the findings in our analysis of the quantile treatment effects of the MNP intervention on child anemia prevalence, which confirms that the more vulnerable children were at baseline (in terms of iron-deficiency), the more benefits were (on average) earned from the intervention. Policy makers need to pay more attention to those children that are at risk of the anemia and take actions to increase their levels of Hb as early as possible.

This study makes several key contributions based on the use of an MNP intervention to improve the anemic status of young children in the medium term. First, unlike other studies that have examined only the short-term impact of an MNP intervention on the anemia of young children, this is the first study to provide evidence of the medium-term impact of an MNP intervention on the anemic status of children in rural China. Our study offers important insights into the role that an MNP intervention can play in improving the anemic status of young children in low-income rural areas of China in the medium term. This is particularly important for developing countries in which children suffer from anemia, which may result in developmental delays. Second, this study uses the differences between children in treatment and control groups as part of a randomized control trial to measure the impact of the intervention on the anemia of young children in rural China; therefore, we can draw a causal conclusion that the MNP intervention can reduce the prevalence of anemia of children in rural China. Third, this study is an effectiveness trial designed to mimic the medium-term impact of a government program. Our results also offer insights into how policymakers can improve children’s anemic status and support human capital accumulation.

Despite its strengths, this study has several limitations. First, there was a relatively high attrition rate (19%) in the follow-up study. Fortunately, the rates of attrition were similar in the treatment and control groups. Hence, the results were likely not affected substantially, as it is plausible that the factors that drove attrition are the same across the groups. Second, we were unable to collect health/nutrition information on any other set of biomarkers beyond hemoglobin. Other indicators, such as each child’s iron status, markers of inflammation, and/or additional mineral or vitamin biomarkers, would have been useful. Third, this study did not conduct any encouragement mechanisms regarding the uptake of the study’s MNP sachets, thus we are unable to measure (and report on) effective ways to increase uptake. Seeking ways to improve the uptake rates should be encouraged in the future studies. Fourth, although our dataset covers a large area of Western China, the sample does not include the many other rural areas in China. As a consequence, the results may not be generalizable to other settings. Therefore, there is a need for further research to be conducted in these areas of rural China.

## Conclusions

Our study provided evidence of one mechanism by which the MNP intervention appears to affect the health outcomes of rural children—by decreasing the rate of the prevalence of anemia and improving the child’s dietary diversity in the medium term. The results in this study found that when using an ITT analysis, four years after the start of the MNP intervention, there was a significant reduction in the prevalence of child anemia and a significant rise in the dietary diversity for children in the treatment group. Moreover, the ATT analysis showed that the intervention significantly reduces the prevalence of child anemia status. The results of quantile treatment effects analysis also showed that children who initially have the most severe anemia levels benefit more from the MNP intervention. The findings of the current study reveal that the MNP intervention has medium-term effects on the nutritional status of children in rural China. The impacts of the MNP program were relatively higher for children that initially had more severe anemia levels. Hence, the implications of this study are that programs that aim to increase caregiver knowledge of nutrition and improve their feeding practices should be encouraged across rural China. Families, policymakers, and China’s society overall need to continue to pay more attention to problems of childhood anemia in rural areas. This is particularly crucial for families with moderately anemic children at an early age as it can significantly contribute to improving the anemia status of children across rural areas of China.

### Supplementary Information

**Additional file 1:** **Table S1.** Comparison of the variables between the treatment and control groups within remained sample at baseline. **Table S2.** Comparison of the variables between the treatment and control groups within remained sample. **Table S3.** The number of sachets consumed in treatment group. **Table S4.** Distribution of the number of sachets consumed in treatment group. **Table S5.** Heterogeneous of MNP program treatment effects on child anemia prevalence, 18 months after the start of the intervention. **Table S6.** Heterogeneous of MNP program treatment effects on child hemoglobin concentration, 18 months after the start of the intervention. **Table S7.** Heterogeneous of MNP program treatment effects on child minimum dietary diversity, 18 months after the start of the intervention. **Table S8.** Heterogeneous of MNP program treatment effects on child dietary diversity index, 18 months after the start of the intervention. **Table S9.** Heterogeneous of MNP program treatment effects on child calorie index, 18 months after the start of the intervention.

## Data Availability

The datasets used and/or analyzed during the current study are available from the corresponding author on reasonable request.

## Abbreviations

MNP: Multiple micronutrient powders
Hb: Hemoglobin
ITT: Intention-to-treat
ATT: Average treatment effect on the treated
WHO: World Health Organization
IQ: Intelligence Quotient
RCT: Randomized controlled trial (RCT)
GDP: Gross domestic product
CDC: Centers for Disease Control and Prevention
IV: Instrumental variable
QTE: Quantile treatment effects
CI: Confidence interval
