# Supplementary material for: The medium-term impact of a micronutrient powder intervention on anemia among young children in Rural China
Source: BMC Public Health. 2024 Feb 10;24:426. doi: 10.1186/s12889-024-17895-2 (PMC10858501; doi:10.1186/s12889-024-17895-2)
Supplement: Supplementary file 1 — Additional file 1: Table S1. Comparison of the variables between the treatment and control groups within remained sample at baseline. Table S2. Comparison of the variables between the treatment and control groups within remained sample. Table S3. The number of sachets consumed in treatment group. Table S4. Distribution of the number of sachets consumed in treatment group. Table S5. Heterogeneous of MNP program treatment effects on child anemia prevalence, 18 months after the start of the intervention. Table S6. Heterogeneous of MNP program treatment effects on child hemoglobin concentration, 18 months after the start of the intervention. Table S7. Heterogeneous of MNP program treatment effects on child minimum dietary diversity, 18 months after the start of the intervention. Table S8. Heterogeneous of MNP program treatment effects on child dietary diversity index, 18 months after the start of the intervention. Table S9. Heterogeneous of MNP program treatment effects on child calorie index, 18 months after the start of the intervention. [file 12889_2024_17895_MOESM1_ESM.docx]

**Supplementary material**

**Table S1. Comparison of the variables between the treatment and control groups within remained sample at baseline**

|  | Treatment group | Control group | *p* value |
| --- | --- | --- | --- |
| Variable | (1) | (2) | (3) |
| ***Child characteristics*** |  |  |  |
| Age | 9.44 | 9.45 | 0.882 |
| (in months) | (1.80) | (1.89) |  |
| Male | 0.53 | 0.49 | 0.098 |
| (1 = yes) | (0.50) | (0.50) |  |
| Low birth weight | 0.07 | 0.06 | 0.424 |
| (1 = yes) | (0.25) | (0.23) |  |
| ***Child anemic status*** |  |  |  |
| Hemoglobin concentration | 109.26 | 109.43 | 0.798 |
| (g/L) | (12.07) | (12.05) |  |
| Anemia prevalence | 0.49 | 0.51 | 0.516 |
| (1 = yes) | (0.50) | (0.50) |  |
| **Attrition rate (%)** | 18.88 | 18.52 | 0.857 |
| **Observations** | 967 | 497 |  |

**Table S2. Comparison of the variables between the treatment and control groups within remained sample**

|  | Treatment (1 = yes) | |
| --- | --- | --- |
| Variable | (1) | (2) |
| ***Child characteristics*** |  |  |
| Age | -0.003 | -0.002 |
| (in months) | (0.008) | (0.009) |
| Male | 0.046* | 0.046 |
| (1 = yes) | (0.025) | (0.031) |
| Low birth weight | 0.046 | 0.097* |
| (1 = yes) | (0.047) | (0.057) |
| ***Child anemic status*** |  |  |
| Hemoglobin concentration | -0.002 | -0.002 |
| (g/L) | (0.002) | (0.002) |
| Anemia prevalence | -0.054 | -0.053 |
| (1 = yes) | (0.042) | (0.052) |
| ***Child dietary diversity*** |  |  |
| Minimum dietary diversity | 0.029 | -0.026 |
| (1 = 4 or above 4 food groups) | (0.045) | (0.057) |
| Dietary diversity index | -0.036 | -0.011 |
|  | (0.027) | (0.031) |
| Calorie index | 0.032 | 0.017 |
|  | (0.020) | (0.023) |
| **Observations** | 1,464 | 1,069 |

*Note.* We adjusted for cohort and county fixed effects, and standard errors are clustered at village level.

**p* < 0.1; ***p* < 0.05; ****p* < 0.01

**Table S3. The number of sachets consumed in treatment group**

| Intervention time | MNP group |
| --- | --- |
| First 6 months of intervention | 66.35 |
|  | (50.53) |
| Between the 7^th^ month and the 12^th^ month of intervention | 89.02 |
|  | (64.72) |
| Between the 13^th^ month and the 18^th^ month of intervention | 81.10 |
|  | (69.18) |
| Average | 236.47 |
|  | (150.72) |

*Note.* Each child in treatment group should eat 540 sachets in total in the MNP intervention.

**Table S4. Distribution of the number of sachets consumed in treatment group**

| Percentage | Percentage (%) |
| --- | --- |
| < 10% (54 sachets ) | 17.83 |
| < 20% (108 sachets ) | 26.57 |
| < 30% (162 sachets ) | 33.04 |
| < 40% (216 sachets ) | 44.58 |
| < 50% (270 sachets ) | 54.55 |
| < 60% (324 sachets ) | 66.43 |
| < 70% (378 sachets ) | 78.15 |
| < 80% (432 sachets ) | 88.99 |
| < 90% (486 sachets) | 97.20 |
| < 100% (540 sachets) | 100 |

*Note.* Each child in treatment group should eat 540 sachets in total in the whole MNP intervention.

**Table S5. Heterogeneous of MNP program treatment effects on child anemia prevalence, 18 months after the start of the intervention**

|  |  | Anemia prevalence (1 = yes) | | |
| --- | --- | --- | --- | --- |
|  |  | Hb < 100 | Hb < 90 | Hb < 80 |
| Variable | | (1) | (2) | (3) |
| (1) | MNP treatment | -0.00 | 0.00 | 0.00 |
|  | (1 = yes) | (0.03) | (0.03) | (0.03) |
| (2) | Covariate | 0.04 | 0.11 | 0.34** |
|  |  | (0.04) | (0.08) | (0.15) |
| (3) | Interaction | 0.01 | -0.10 | -0.27 |
|  |  | (0.06) | (0.09) | (0.19) |
| (4) | Observations | 1,403 | 1,403 | 1,403 |
| Test the long-term effects of the treatment on child anemia prevalence with different Hb levels | | | | |
| (5) | Effect of the treatment with covariate samples | 0.01 | -0.09 | -0.27 |
| (6) | *p*-value | 0.94 | 0.28 | 0.16 |

*Note.* Each column is a separate regression. Covariate represents Hb < 100, Hb < 90, and Hb < 80. Interaction represents treatment * Hb <100, treatment * Hb < 90, and treatment * Hb < 80. Controls include the baseline value of the outcome variable, child’s age, gender, and whether the child had a low birth weight. We adjusted for cohort and county fixed effects, and standard errors are clustered at village level.

**p* < 0.1; ***p* < 0.05; ****p* < 0.01

**Table S6. Heterogeneous of MNP program treatment effects on child hemoglobin concentration, 18 months after the start of the intervention**

|  |  | Hemoglobin concentration (g/L) | | |
| --- | --- | --- | --- | --- |
|  |  | Hb < 100 | Hb < 90 | Hb < 80 |
| Variable | | (1) | (2) | (3) |
| (1) | MNP treatment | 0.17 | -0.31 | -0.33 |
|  | (1 = yes) | (0.86) | (0.81) | (0.79) |
| (2) | Covariate | -0.52 | -3.31 | -9.28*** |
|  |  | (1.35) | (2.53) | (3.48) |
| (3) | Interaction | -1.75 | 2.30 | 7.92* |
|  |  | (1.73) | (3.02) | (4.25) |
| (4) | Observations | 1,403 | | |
| Test the long-term effects of the treatment on child anemia prevalence with different Hb levels | | | | |
| (5) | Effect of the treatment with covariate samples | -1.58 | 1.99 | 7.59* |
| (6) | *p*-value | 0.33 | 0.50 | 0.07 |

*Note.* Each column is a separate regression. Covariate represents Hb < 100, Hb < 90, and Hb < 80. Interaction represents treatment * Hb <100, treatment * Hb < 90, and treatment * Hb < 80. Controls include the baseline value of the outcome variable, child’s age, gender, and whether the child had a low birth weight. We adjusted for cohort and county fixed effects, and standard errors are clustered at village level.

**p* < 0.1; ***p* < 0.05; ****p* < 0.01

**Table S7. Heterogeneous of MNP program treatment effects on child minimum dietary diversity, 18 months after the start of the intervention**

|  |  | Minimum dietary diversity (1 = 4 or more than 4 food groups) | | |
| --- | --- | --- | --- | --- |
|  |  | Hb < 100 | Hb < 90 | Hb < 80 |
| Variable | | (1) | (2) | (3) |
| (1) | MNP treatment | 0.04 | 0.03 | 0.03 |
|  | (1 = yes) | (0.03) | (0.03) | (0.03) |
| (2) | Covariate | 0.05 | -0.00 | -0.24 |
|  |  | (0.05) | (0.08) | (0.18) |
| (3) | Interaction | -0.03 | 0.07 | 0.29 |
|  |  | (0.07) | (0.10) | (0.22) |
| (4) | Observations | 1,451 | | |
| Test the long-term effects of the treatment on child anemia prevalence with different Hb levels | | | | |
| (5) | Effect of the treatment with covariate samples | 0.01 | 0.10 | 0.32 |
| (6) | *p*-value | 0.83 | 0.32 | 0.16 |

*Note.* Each column is a separate regression. Covariate represents Hb < 100, Hb < 90, and Hb < 80. Interaction represents treatment * Hb <100, treatment * Hb < 90, and treatment * Hb < 80. Controls include the baseline value of the outcome variable, child’s age, gender, and whether the child had a low birth weight. We adjusted for cohort and county fixed effects, and standard errors are clustered at village level.

**p* < 0.1; ***p* < 0.05; ****p* < 0.01

**Table S8. Heterogeneous of MNP program treatment effects on child dietary diversity index, 18 months after the start of the intervention**

|  |  | Dietary diversity index | | |
| --- | --- | --- | --- | --- |
|  |  | Hb < 100 | Hb < 90 | Hb < 80 |
| Variable | | (1) | (2) | (3) |
| (1) | MNP treatment | -0.01 | -0.02 | -0.00 |
|  | (1 = yes) | (0.06) | (0.05) | (0.05) |
| (2) | Covariate | -0.12 | -0.21 | -0.60* |
|  |  | (0.11) | (0.15) | (0.32) |
| (3) | Interaction | 0.12 | 0.37* | 0.64 |
|  |  | (0.13) | (0.20) | (0.41) |
| (4) | Observations | 1,451 | | |
| Test the long-term effects of the treatment on child anemia prevalence with different Hb levels | | | | |
| (5) | Effect of the treatment with covariate samples | 0.10 | 0.35* | 0.63 |
| (6) | *p*-value | 0.35 | 0.06 | 0.13 |

*Note.* Each column is a separate regression. Covariate represents Hb < 100, Hb < 90, and Hb < 80. Interaction represents treatment * Hb <100, treatment * Hb < 90, and treatment * Hb < 80. Controls include the baseline value of the outcome variable, child’s age, gender, and whether the child had a low birth weight. We adjusted for cohort and county fixed effects, and standard errors are clustered at village level.

**p* < 0.1; ***p* < 0.05; ****p* < 0.01

**Table S9. Heterogeneous of MNP program treatment effects on child calorie index, 18 months after the start of the intervention**

|  |  | Calorie index | | |
| --- | --- | --- | --- | --- |
|  |  | Hb < 100 | Hb < 90 | Hb < 80 |
| Variable | | (1) | (2) | (3) |
| (1) | MNP treatment | -0.03 | -0.01 | -0.02 |
|  | (1 = yes) | (0.06) | (0.06) | (0.05) |
| (2) | Covariate | 0.08 | 0.12 | -0.18 |
|  |  | (0.11) | (0.16) | (0.35) |
| (3) | Interaction | 0.06 | -0.08 | 0.14 |
|  |  | (0.13) | (0.21) | (0.42) |
| (4) | Observations | 1,464 | | |
| Test the long-term effects of the treatment on child anemia prevalence with different Hb levels | | | | |
| (5) | Effect of the treatment with covariate samples | 0.03 | -0.09 | 0.13 |
| (6) | *p*-value | 0.75 | 0.66 | 0.76 |

*Note.* *Note.* Each column is a separate regression. Covariate represents Hb < 100, Hb < 90, and Hb < 80. Interaction represents treatment * Hb <100, treatment * Hb < 90, and treatment * Hb < 80. Controls include the baseline value of the outcome variable, child’s age, gender, and whether the child had a low birth weight. We adjusted for cohort and county fixed effects, and standard errors are clustered at village level.

**p* < 0.1; ***p* < 0.05; ****p* < 0.01
